# Development of Novel Foxtail Millet-Based Nutri-Rich Instant Noodles: Chemical and Quality Characteristics

**DOI:** 10.3390/foods12040819

**Published:** 2023-02-14

**Authors:** Mst. Meherunnahar, Tanvir Ahmed, Razia Sultana Chowdhury, Mohammed Abdus Satter Miah, Kandi Sridhar, Baskaran Stephen Inbaraj, Md. Mozammel Hoque, Minaxi Sharma

**Affiliations:** 1 Department of Food Engineering and Tea Technology, Shahjalal University of Science and Technology, Sylhet 3100, Bangladesh; 2 Institute of Food Science and Technology (IFST), Bangladesh Council of Scientific and Industrial Research (BCSIR), Dhaka 1205, Bangladesh; 3 Department of Food Technology, Karpagam Academy of Higher Education Deemed to be University, Coimbatore 641021, India; 4 Department of Food Science, Fu Jen Catholic University, New Taipei City 242062, Taiwan; 5 Department of Applied Biology, University of Science and Technology, Meghalaya 793101, India

**Keywords:** noodles, composite flour, foxtail millet, wheat, mushroom, amino acids

## Abstract

Noodles are a popular snack mainly produced from wheat flour; however, the low contents of protein, minerals, and lysine are a concern. Therefore, this research developed nutri-rich instant noodles by using foxtail millet (FTM) (*Setaria italic*) flour to improve the contents of protein and nutrients and increase its commercial importance. FTM flour was mixed with wheat flour (*Triticum aestivum*) at a ratio of 0:100, 30:60, 40:50, and 50:40, and the samples were named as control, FTM30, FTM40, and FTM50 noodles, respectively. Mushroom (*Pleurotus ostreatus*) and rice bran (*Oryza sativa* L.) flour were added at a percentage of 5% to all the composite noodles (FTM30, FTM40, and FTM50 noodles). The contents of biochemicals, minerals, and amino acids, as well as the organoleptic properties of the noodles, were examined and compared with wheat flour as a control. The results revealed that the carbohydrate (CHO) content of FTM50 noodles was significantly lower (*p* < 0.05) than all the developed and five commercial noodles named A-1, A-2, A-3, A-4, and A-5. Moreover, the FTM noodles had significantly higher levels of protein, fiber, ash, calcium, and phosphorous than the control and commercial noodles. The percentage of lysine calculated protein efficiency ratio (PER), essential amino acid index (EAAI), biological value (BV), and chemical score (CS) of FTM50 noodles were also higher than that of the commercial noodles. The total bacterial count was nil for the FTM50 noodles, and the organoleptic properties were consistent with those of acceptable standards. The results could encourage the application of FTM flours for the development of variety and value-added noodles with enhanced level of nutrients.

## 1. Introduction

Noodles are a convenient meal because of their ease of preparation, low cost, and relatively long shelf life. Changing food habits, increasing population, and urbanization have led to increasing consumption of noodles worldwide. It is mainly produced from wheat flour, which contains 10–12% protein. Wheat (*Triticum aestivum*) is the second major food in Bangladesh and is extensively used in bakery and confectionery products. However, the local climatic conditions in Bangladesh are not very conducive for wheat farming. In Bangladesh, the consumption rate of wheat is 7.1 million metric tons (mmt) per year, whereas production is only 1.15 mmt per year [1]. Therefore, Bangladesh imports the deficit quantity to satisfy consumer demand every year, thereby posing an economic threat to the baking industry.

Foxtail millet (FTM) (*Setaria italica*) is a drought-tolerant and salt-tolerant crop with little use of herbicides for crop growth. Though Bangladesh FTM production fluctuated substantially in recent years, it tended to increase through 1972–2021 period, ending at 9616.28 tonnes in 2021 [2]. Therefore, it is currently receiving national and international interest. FTM is a non-glutinous, nonacid-forming, and easy-to-digest food that is high in energy and protein and can be cultivated in a variety of agro-climatic conditions. Bangladesh is located in the largest deltaic land in the world, and a large portion of it remains uncultivable. Moreover, food security is threatened by rising sea levels, loss of fertile lands, frequent floods, and severe weather patterns. Therefore, growing FTM in abandoned or unproductive soil could be a major solution for such places. FTM adapts well to climate changes and can be grown in semiarid or arid regions where wheat or other crops cannot be cultivated. The nutritional profile of FTM is also superior to the staple cereals (rice and wheat), and possess comparable contents of protein (10–14%), calcium (20–30 mg/100 g), iron (5–7 mg/100 g), phosphorous (500–600 mg/100 g), and fiber (7–8%) [3]. It acts as a binding agent in the production of high-calorie foods. The use of FTM flour in combination with wheat flour may result in the production of food items that may enhance national food security.

Mushrooms (*Pleurotus ostreatus*) are simple plant leaves found throughout the world and are a source of nutrition and medicine. It is deficient in chlorophyll, fat, and calories but rich in proteins, vitamins, minerals, folic acids, and dietary fibers. Mushrooms are cooked with other foods to increase their nutritive value, or they are used as a strengthening agent with other herbs [4]. Furthermore, Rice bran *(Oryza sativa* L.) is the outer layer of the rice kernel, a byproduct of the milling system, and is a rich source of antioxidants that reduce blood cholesterol and decrease the incidence of atherosclerosis. Rice bran is a rich source of proteins, fiber, vitamins (vitamins B1 and B2), minerals (Iron, Mn, Mg, etc.), and bioactive compounds (oryzanol, tocopherol, and tocotrienol) [5]. Many researchers have focused on rice bran and called it a functional superfood. Therefore, mushrooms and rice bran can be used as supplements to improve the nutritional status of baked products.

Bangladesh is the most densely populated country in the world, and approximately 25% of people are living in deep poverty and food insecurity. Bangladeshis follow an imbalanced diet that is dominated by cereals. Rising sea levels, loss of arable lands, frequent flooding, extreme weather patterns, and the COVID-19 pandemic pose threats to food security. Therefore, children and women in Bangladesh have extreme malnutrition and micronutrient deficiencies. However, people are becoming health conscious, and several researchers are focusing on the diversified use of multigrain flour to improve the nutritional quality of daily foods. In Bangladesh, composite flour-based noodles are unavailable to maintain a balanced diet. Consequently, this study developed highly nutritive instant noodles from FTM-based composite flour (FTM, wheat, mushroom, and rice bran flour) to improve the condition of malnutrition and reduce the consumption of wheat flour.

## 2. Materials and Methods

### 2.1. Material Collection

FTM was collected from Plant Breeding Division, Bangladesh Agricultural Research Institute, Joydebpur, Gazipur, Bangladesh. Wheat was collected from Bangladesh Wheat Research Institute, Dinajpur, Bangladesh; mushroom was collected from National Mushroom Development and Extension Center, Savar, Bangladesh; and rice bran was collected from Bangladesh Agricultural Development Corporation, Sylhet, Bangladesh. The best-quality local commercial noodles were collected from supermarkets in Dhaka city to compare their nutritional quality with that of the prepared noodles.

### 2.2. Composite Flour

Whole wheat flour was mixed with FTM flour, mushroom, and rice bran flour at a ratio of 100:0:0:0, 60:30:5:5, 50:40:5:5, and 40:50:5:5 to obtain four types of flour, namely control, FTM30, FTM40, and FTM50, respectively.

### 2.3. Noodles Preparation

Noodles were prepared as described by Collins and Pangloli (1997). The ingredients for each composite flour were weighed accurately and mixed properly for dough preparation. The dough was rounded, covered with wrapping paper for fermentation at an ambient temperature, and allowed to stand for 30 min. Subsequently, it was kneaded by hand for 1 min and sheeted using a pasta machine (ATLAS 150 WELL.AS.P, Marcato, Padova, Italy). Then, the sheets were placed on a hand-operated pasta machine to obtain 3 mm noodle threads. The threads were transferred on a net for steaming at a temperature of 100 °C for 3 min. The steamed noodles were dried in a food-grade dryer (TB-30-ADFP, TAIKI SANGYO Co., Ltd., Okayama, Japan) at 70 °C for 6–8 h. The dried noodles were cooled at room temperature, packed in an airtight plastic bag, and stored at 22–25 °C for further analysis.

### 2.4. Proximate Analysis

The contents of moisture, crude protein, fat, ash, carbohydrate, and crude fiber in the prepared flours were determined using the Approved Methods of Official Analytical Chemists [6]. The total energy value was calculated as per Yu’s method [7] by using the following formula according to Equation (1):

(1) $$\mathrm{Energy}\;\left(\frac{\mathrm{Kcal}}{100}g\right)=\left[\left(\%\mathrm{Carbohydrates}\times 4.1\right)+\left(\%\;\mathrm{Protein}\times 4.1\right)+\left(\%\mathrm{fat}\times 9.3\right)\right].$$

The carbohydrate content (%) was evaluated as follows, according to Equation (2):

(2) Carbohydrate content (%)=[100−(% water+% fat+% protein+% ash)].

### 2.5. Mineral Analysis

Mineral contents were determined using the Official Method of the American Association of Cereal Chemists (AACC) [8]. Samples were dried and ashed at 700 °C for 6 h. The ash was dissolved in 3 mL hydrochloric acid and 5 mL distilled water; the final volume of 50 mL was achieved using distilled water, and the solution was filtered. The contents of sodium (y = 0.389x + 0.36, |r| = 0.99) and potassium (y = 0.364x + 0.375, |r| = 0.97) were determined using flame photometry (PFP7, LABEQUIP LTD., Markham, Canada). The contents of calcium (y = 0.0004x + 0.0017, |r|= 0.98), iron (y = −0.0006x + 0.0052, |r| = 0.78), and phosphorous (y = 0.0869x − 0.0055, |r| = 0.99) were determined using the atomic absorption spectrometer (Model AA-670G V-5, Shimadzu, Kyoto, Japan).

### 2.6. Microbial Analysis

A microbiological examination of the noodles was performed to assess bacterial, fungal, yeast, and mold counts under laboratory conditions. Standard Plate Count (SPC), fungal, yeast, and mold counts, and total counts of coliform bacteria and Salmonella spp. in the noodles were determined using the method of the American Public Health Association [9]. The plate count method was employed for the determination of the total number of viable microbes in the noodles. SPC was estimated using the decimal dilution technique, followed by pour plate and spread plate methods for fungus and yeast, respectively. The streak plate method was used to isolate the specific microorganism. Isolation and enumeration of total coliform bacteria were performed using the most probable number method [10] and by using Mac Conkey broth (125PP-1900-100, General laboratory Products, Yorkville, IL, USA).

### 2.7. Organoleptic Evaluation

Organoleptic evaluation of the prepared noodles was performed using the method of Chauhan et al. [11]. A 7-point hedonic scale was used to evaluate the nutritional attributes by 15 trained taste panel referees of Cereal Technology Laboratory, Bangladesh Council of Scientific and Industrial Research, Dhaka 1205, Bangladesh. Appearance, color, texture, flavor, taste, and overall acceptability of the prepared noodles were evaluated. The scale was scored as “like extremely”, “like very much”, “like moderately”, “like slightly”, “neither like or dislike”, “dislike very much”, and “dislike extremely”. The quality attributes of noodles prepared from the composite flours were evaluated and compared with those of control and commercial noodles.

### 2.8. Amino Acid Analysis

The protein quality of FTM noodles was determined on the basis of the amino acid profiles that were determined using the Technicon Sequential Multi-Sample Amino Acid Analyzer (Technicon instrument corporation, Dublin, Ireland). The samples were hydrolyzed for 22 h at 105 ± 5 °C with 6 M HCL as per the method of Jamroz et al. [12]. Amino acid score, calculated protein efficiency ratio (C-PER), predicted protein efficiency ratio (PER), essential amino acid index (EAAI), and biological value were determined to evaluate the nutritional quality of the prepared FTM noodles. The amino acid composition of the whole egg protein and the Food and Agriculture Organization/World Health Organization [13] reference amino acid pattern were used as standards for calculating the chemical score according to Equation (3).

(3) $$\mathrm{Chemical}\;\mathrm{score}\;\left(\mathrm{CS}\right)=\left[\left(\frac{\mathrm{Test}\;\mathrm{amino}\;\mathrm{acid}}{\mathrm{Amino}\;\mathrm{acid}\;\mathrm{standard}}\right)\times 100\right].$$

The calculated protein efficiency ratio (C-PER) was analyzed using Equation (4) developed by Hidvégi and Békés [14].

(4) C−PER=[−1.816+0.435 (Methio)+0.780 (Leu)+0.211 (His)−0.944 (Tyro)].

The predicted protein efficiency ratio (P-PER) values of the different accessions were calculated from their amino acid composition by using the regression Equation (5) developed by Hidvégi and Békés [14].

(5) P−PER=[0.486+0.454 (Leu)−0.105 (Tyro)].

The Essential Amino Acid Index (EAAI) was calculated according to the method of Zinina et al. [15] and by using the following Equation (6):

(6) $$\mathrm{EAAI}=\left[\left(n\sqrt{\frac{100a\times 100b\ldots 100j}{\mathrm{av}\times \mathrm{bv}\ldots \mathrm{jv}}}\right)\right],$$

where n = number of essential amino acids, a, b …j = the concentration of essential amino acids (lysine, tryptophan, isoleucine, valine, arginine, threonine, leucine, phenylalanine, histidine, and the sum of methionine and cysteine) in the test sample and av, bv … jv = content of the same amino acids in standard protein percent (Egg or casein), respectively. The biological value was calculated as described by Kaur et al. [16] and by using the following Equation (7):

(7) BV=[(1.09×EAAI)].

### 2.9. Statistical Analysis

Data were analyzed using a one-way analysis of variance, and the Tukey honestly significant difference test was applied for the comparison of each group. Statistical analyses were performed using SPSS (IBM SPSS 22.0, Chicago, IL, USA). Data are presented as mean ± standard deviation. The *p* values of <0.05 were considered statistically significant.

## 3. Results and Discussion

### 3.1. Evaluation of Biochemical Properties of FTM Noodles

The biochemical properties of the prepared noodles are listed in Table 1. Significant differences (*p* < 0.05) were observed between the prepared and control noodles. FTM50 noodles had a significantly higher (*p* < 0.05) moisture content (6.61%) than the control noodles (5.11%). Moisture is the most crucial element affecting food preservation and the shelf life of grain ingredients. Moreover, FTM50 noodles had the highest ash content (2.61%); the presence of more inorganic nutrients in the noodles may be responsible for the dark color [17]. The fat content of the prepared noodles varied from 14.32% to 17.85%. FTM50 noodles contained significantly higher (*p* < 0.05) fat content (17.85%) than the control (14.32%), FTM30 (16.51%), and FTM40 (16.23%) noodles. Although the same quantity of fat was used in the preparation of the noodles, a variation was observed due to the use of different proportions of wheat and FTM flour. The fiber content was enhanced by using FTM flour instead of regular flour, with the content varying from 2.42 to 5.37%. A higher percentage of fiber was observed in the FTM50 noodles (5.37%) compared with the control noodles (2.42%). The noodles prepared entirely from wheat flour had the lowest percentage of protein (10.75%), whereas FTM50 noodles had a considerably higher (*p* < 0.05) percentage of protein (14.78%) because of the use of a higher percentage of FTM. The FTM50 noodles are an excellent source of dietary protein and may fulfill the daily protein requirements for humans. Moreover, the FTM50 noodles contained significantly lower (*p* < 0.05) percentages of carbohydrates (57.74%) than the control noodles (67.07%). Carbohydrates are organic compounds that serve as a source of energy. The low carbohydrate content in the FTM noodles has several health benefits, such as improvement of digestion in the colon and reduction in constipation from refined grain flours [18].

### 3.2. Organoleptic Properties of FTM Noodles

**Figure 1:**
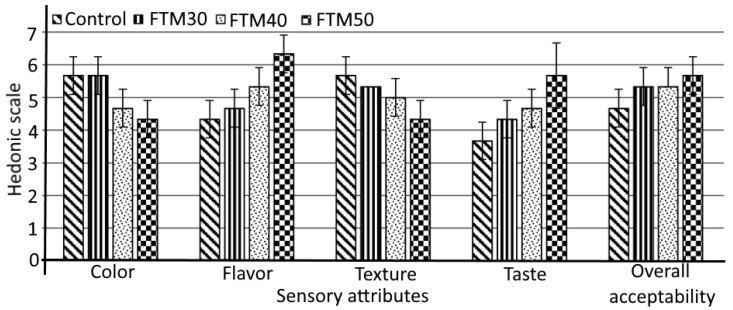
Sensory attributes of foxtail millet noodles. Results are expressed as mean values of 15 independent determinations. Mean values followed by error bars represent the standard deviation from the mean value. FTM, foxtail millet. For sample codes, refer to Table 1.

The organoleptic score of the FTM noodles is displayed in Figure 1. The color of the noodles changed with the substitution of FTM flour. The noodles prepared entirely from wheat flour had the best color score value (5.67), which decreased with increasing levels of FTM flour. The lowest color score value was observed for the FTM50 noodles (4.33). Variation in flavor and taste was observed among the noodles. The FTM50 noodles received a taste score of 5.67, which was significantly higher (*p* < 0.05) than those of the control (3.67), FTM30 (4.33), and FTM40 noodles (4.67), respectively. The incorporation of FTM and other components (5% mushroom and 5% rice bran flour) enhanced the flavor score of the FTM50 noodles (6.33) compared with the control noodles (4.33). The texture of noodles was also affected because of the increasing level of FTM flour substitution. The texture value was significantly higher (*p* < 0.05) in the control noodles (5.67) than in the FTM noodles (ranging from 5.00 to 4.33). Because FTM is a hardy crop, the preparation of dough was challenging. Moreover, strands of noodles tend to break due to the hardness if more than 50% of FTM flour is used [19]. No significant variation (*p* < 0.05) in overall acceptability was observed among the FTM30, FTM40, FTM50, and control (100% wheat flour) noodles. Therefore, the acquired organoleptic scores confirmed the improvement of FTM noodles in terms of flavor and taste. The improved flavor and taste could be related to the dough fermentation. The results indicate that various forms of noodles and baked products can be produced using up to 50% of FTM flour. This may enrich the nutrition of a product and reduce the consumption of wheat flour. These results are consistent with those of Chillo et al. [20].

### 3.3. Evaluation of Amino Acids in FTM Noodles

The nutritional quality of proteins depends primarily on the capacity of nitrogen and its essential amino acids. For the maintenance of excellent health and regular functioning of the human body, 20 distinct amino acids are required [21]. Of these, nine amino acids are essential, and the FTM noodles contained eight of these essential amino acids (EAA). The amino acid composition of the FTM noodles and their nutritional values, CS, C-PER, P-PER, EAAI, and BV, are presented in Table 2. The results indicated that mixing FTM flour with wheat flour improved the amino acid profile. Lysine, especially, increased from 5.6% to 11%, and threonine increased from 3.7% to 5.4% in the FTM50 noodles. The total essential amino acid (TEAA) values of the FTM and control noodles ranged from 30.1 to 45.7%; these values are consistent with those of Eman et al. [22], who reported the levels of amino acids in wheat flour, lupine, and their mixtures as 35.91–36.10%. However, the CS of the control noodles (91.77%) increased gradually with the increase in FTM, with the maximum CS obtained in the FTM50 noodles (139.33%). The C-PER increased to its maximum level of 1.67% in the FTM30 noodles. The control noodles prepared from 100% wheat flour had the lowest C-PER value of 0.60%. The P-PER of the samples was 1.71%, 2.26%, 2.38%, and 2.28% in the control, FTM30, FTM40, and FTM50 noodles, with the highest value of P-PER observed in the FTM40 noodles (2.38%) and the lowest value in the control noodles (1.71%). The EAAI value ranged from 68.57 to 95.02% in the control and FTM50 noodles. The control noodles had the lowest EAAI score (68.57%), whereas the highest level of EAAI value was observed in the FTM50 noodles (95.02%). The protein content of a food material is considered optimal if the EAAI value is above 90%. If the EAAI value is below 70%, the protein content is considered inadequate [23]. Oser [23] also reported that if the BV of food is in a high range (70–100%), the food is considered nutritious. The FTM50 noodles had the highest level of BV (91.87%), followed by the FTM30 (90.41%), FTM40 (86%), and control (63.04%) noodles. The incorporation of FTM flours into wheat flour considerably improved the protein quality of the noodles. The addition or substitution of raw materials rich in proteins resulted in noodles with higher protein contents and better nutritional values than conventional ones.

### 3.4. Evaluation of Microbial Quality in FTM Noodles

The counts of microorganisms in the FTM noodles during the 6-month storage period are listed in Table 3. The results indicated that the counts of total viable bacteria, yeast, and mold were within acceptable levels. The count of coliform and the presence of *salmonella* and *bacillus* per gram of the FTM noodles were nil/g. The low counts of an organism in prepared foods indicate the good quality of the thermal process, raw materials, and processing conditions under which the foods are produced [24].

### 3.5. Nutrient Content of FTM50 Noodles with Respect to Recommended Dietary Allowance

Nutrient intake at an adequate level is required for a healthy lifestyle. The percentage of nutrients per 100 g of the FTM50 noodles could fulfill the Recommended Dietary Allowance (RDA) (Table 4). In terms of the RDA for men, the protein density per 100 g of the FTM50 noodles would meet approximately 32.84%, 25.05%, 25.48%, and 23.46% of the RDA for the age groups of 11–14, 15–18, 19–24, and 25–51+ years, respectively, with energy recovery of 18.58, 15.48, 16.02, and 20.20 kcal/100 gm, respectively. For women, the FTM50 noodles would meet 32.13%, 33.59%, 29.56%, 24.63%, 22.73%, and 23.83% of the RDA for the age groups of 11–14 years, 19–24 years, 15–18 years, 25–51+ years, pregnancy, first 6 months of lactation, and second 6 months of lactation, respectively. According to a study named Desirable Dietary Pattern for Bangladesh by Fennema [25], the extra requirements of protein are 15 g and 19 g during pregnancy and the first six months of the lactation period. The FTM50 noodles will meet 98.27% and 77.57% of the extra protein requirements in pregnant and lactating mothers, respectively. In females, the energy recovery of the FTM noodles can meet 21.11%, 24.45%, 18.58%, and 17.2% of the RDA for those aged 11–50 years, 51+ years, expectant mothers, and nursing mothers, respectively. The extra recommended energy value for pregnant and lactating mothers is approximately 300 kcal and 550 kcal, respectively [26]. The FTM50 noodles will provide 154.87% and 84.47% of the extra energy requirement in urban pregnant mothers with moderate physical activity [27]. Adolescent boys and girls grow faster in their first year of life, thereby requiring extra energy. Therefore, the FTM50 noodles can be used to provide extra energy during the adolescent period. The phosphorous level of the FTM50 noodles can provide approximately half (44.73%) of the daily phosphorous requirement in men, women, and pregnant and lactating mothers (first and second 6 months of lactation). Moreover, the FTM noodles provide 67.09% of the phosphorous requirement in men and women aged 11–24 and 25–51+ years. Iron is a key nutrient in the human diet. The FTM noodles provide 27.89 mg and 50.2 mg of iron for men aged 11–18 years and 19–51 years. For women aged 11–50 years and nursing women, the FTM noodles can provide 33.47 mg/100 g of the RDA of iron. When a woman reaches the age of 51 years, the iron in her body begins to deteriorate. The FTM50 noodles will provide half of the total iron required during this period.

### 3.6. Biochemical Evaluation of FTM50 Noodles and Comparison with Commercial Noodles

According to the proximate analysis, sensory evaluation, microbiological and amino acid evaluation of the prepared noodles, the FTM50 noodles fulfilled the standard quality features. Consequently, the FTM50 noodles were compared with commercial noodles to determine their overall nutritional quality (Table 5). The FTM50 noodles contained a significantly higher (*p* < 0.05) amount of protein (14.78%) than all commercial noodles (ranging from 4.42 to 9.50%). The use of FTM-based composite flour improved the quality of FTM instant noodles; therefore, it can be used to alleviate protein and energy malnutrition in children, thereby improving the growth, repair, and maintenance of their bodies. Proteins act as enzymes and hormones to maintain fluid, electrolyte acid–base balance, and a strong immune system [28]. The human body requires adequate calories for tissue building and energy expenditure. Therefore, the energy and carbohydrate density in food is important. The FTM50 noodles contained a significantly lower (*p* < 0.05) amount of carbohydrates (57.74%) compared with all commercial noodles (ranging from 66.74 to 77.98%). Carbohydrates are organic compounds that are the fundamental source of energy. However, it may cause negative health effects, such as obesity, hypertension, and diabetes. Therefore, the total carbohydrate content in the human diet must be maintained within appropriate limits. The FTM noodles also contained a significantly higher (*p* < 0.05) amount of energy (464.61 kcal) than the commercial noodles (ranging from 422.56 to 460.94) kcal/100 g). The amount of calcium and phosphorus (23.40 and 536.75 mg/100 g) in the FTM50 noodles was significantly higher (*p* < 0.05) than in all the commercial noodles. Calcium is the principal mineral contributing to strong bones and is important for muscle contraction, nerve transmission, and blood clotting [29]. The FTM50 noodles can meet the calcium requirements of the body.

Phosphorous enhances the body’s immune system, thereby reducing the risk of infections and fostering the proper functioning of the organs [30]. The FTM50 noodles contained 5.02 mg/100 g of iron which was significantly higher (*p* < 0.05) than those of A-2 (3.21 mg/100 g), A-3 (1.86 mg/100 g), and A-5 (0.76 mg/100 g) commercial noodles but was significantly lower (*p* < 0.05) than those of A-1 (7.75 mg/100 g) and A-4 (8.56 mg/100 g) commercial noodles. Iron is an essential micronutrient for the synthesis of hemoglobin (an oxygen carrier in the red blood cells), myoglobin (used in muscle contraction), and enzymes and coenzymes [31]. Therefore, the FTM50 noodles are a promising source of protein (14.78%), calcium (23.04 mg/100 g), fiber (5.37%), iron (5.02 mg/100 g), and phosphorous (536.75 mg/100 g). Moreover, the FTM noodles will provide high energy, high nutrition, and low carbohydrate (57.74%) compared with commercial noodles.

## 4. Conclusions

Noodles are a popular snack among all age groups; however, those available in the market are nutritionally poor. In the present investigation, attempts have been made to develop nutrient-rich instant noodles by the addition of FTM, wheat, mushroom, and rice bran flour. Among all the formulation tried, noodle sample prepared from 50:40 flour combination (FTM50) showed a promising source of protein, fiber, and minerals. The CS, BV, C-PER, EAAI, and sensory scores of the FTM50 noodles revealed their high nutritional quality compared to that of the commercial noodles. Furthermore, the total bacterial count was nil for the FTM50 noodles, and the organoleptic properties were consistent with those of acceptable standards. Therefore, the FTM50 noodles can be used as an alternative source of protein to alleviate protein and energy malnutrition. Moreover, FTM can be a potential ingredient in household food preparation (noodles, pasta, bread, biscuits, etc.) and will be preferable for working mothers to prepare food in a short time for tiffin or in-office meetings.

## Figures and Tables

**Table 1:** Biochemical properties of the prepared noodles ^1^.

Nutritional Parameters	Control	FTM30	FTM40	FTM50
Moisture (%)	5.11 ± 0.10 ^c^	6.15 ± 0.07 ^b^	6.27 ± 0.07 ^b^	6.61 ± 0.16 ^a^
Ash (%)	1.56 ± 0.08 ^c^	2.39 ± 0.06 ^b^	2.43 ± 0.08 ^b^	2.61 ± 0.16 ^a^
Fat (%)	14.32 ± 0.11 ^d^	16.51 ± 0.08 ^b^	16.23 ± 0.06 ^c^	17.85 ± 0.08 ^a^
Fiber (%)	2.42 ± 0.04 ^d^	3.31 ± 0.44 ^c^	4.44 ± 0.05 ^b^	5.37 ± 0.04 ^a^
Protein (%)	10.75 ± 0.07 ^d^	14.23 ± 0.09 ^c^	14.47 ± 0.07 ^b^	14.78 ± 0.08 ^a^
Carbohydrate (%)	67.07 ± 0.42 ^a^	61.65 ± 0.63 ^b^	60.83 ± 0.06 ^c^	57.74 ± 0.09 ^d^
Energy (kcal/100 g)	456.85 ± 0.41 ^d^	461.84 ± 0.62 ^c^	463.51 ± 0.29 ^b^	464.61 ± 0.61 ^a^
Calcium (mg/100 g)	15.39 ± 0.03 ^d^	17.42 ± 0.06 ^c^	22.65 ± 0.06 ^b^	23.40 ± 0.02 ^a^
Iron (mg/100 g)	0.73 ± 0.04 ^d^	3.47 ± 0.05 ^c^	4.32 ± 0.04 ^b^	5.02 ± 0.04 ^a^
Phosphorous (mg/100 g)	454.33 ±4.72 ^d^	503.19 ± 2.17 ^c^	514.49 ± 1.87 ^b^	536.75 ± 0.08 ^a^
Sodium (mg/100 g)	15.30 ± 0.14 ^d^	25.85 ± 0.11 ^c^	30.31 ± 0.11 ^a^	28.29 ± 0.43 ^b^
Potassium (mg/100 g)	8.78 ± 0.04 ^d^	11.85 ± 0.09 ^c^	32.93 ± 0.06 ^b^	38.15 ± 0.07 ^a^

^1^ Data presented as mean ± standard deviation. Column with the same superscript letters is not significantly different at *p* < 0.05. Control, 100% wheat flour; FTM30, 30% FTM flour + 60% WF + 5% MF + 5% RBF; FTM40, 40% FTM flour + 50% WF+ 5% MF + 5% RBF; FTM50, 50% FTM flour + 40% WF+ 5% MF + 5% RBF; FTM flour, foxtail millet flour; WF, wheat flour; MF, mushroom flour; RBF, rice bran flour.

**Table 2:** Amino acid composition in the prepared noodles ^1^.

Essential Amino Acids	Control (100% WF)	FTM30 (%)	FTM40 (%)	FTM50 (%)	Std. Whole Egg Protein	FAO/WHO Standard
Histidine	1.80	3	2.90	2.70	2.20	1.90
Isoleucine	4.30	5.70	5.80	6	5.40	2.80
Leucine	5.70	7.10	7.40	7.30	6.60	6.60
Lysine	5.60	10.50	10.80	11	7	5.80
Methionine	2.50	3.60	2.20	3.80	5.70	2.50
Tyrosine	3.70	4.50	4.70	5.20	9.30	6.30
Threonine	3.70	5.40	5.20	5.40	4.70	3.40
Valine	3.40	4.80	5.20	4.30	6.60	3.50
Total	30.10	44.60	44.20	45.70	47.50	32.80
Nutritional value (%)						
CS	91.77	135.98	134.76	139.33	-	-
C-PER	0.60	1.67	1.09	1.19	-	-
P-PER	1.71	2.26	2.38	2.28	-	-
EAAI	68.57	93.68	89.63	95.02	-	-
BV	63.04	90.41	86.00	91.87	-	-

^1^ CS, chemical score; C-PER, calculated protein efficiency ratio; P-PER, predicted protein efficiency ratio; EAAI, essential amino acid index; BV, biological value; EAA, essential amino acids; TEAA, total essential amino acid; FAO, food and agricultural organization; WHO, World Health Organization. For sample codes, refer to Table 1.

**Table 3:** Shelf-life of FTM50 noodles ^1^.

Parameters	Colony Count (CFU/g)							Satisfactory Level
	Months							
	0	1	2	3	4	5	6	
Total plate count (TPC)	Nil	Nil	Nil	Nil	1 × 10^2^	2 × 10^2^	2 × 10^2^	≤2 × 10^2^
Yeast and mold	Nil	Nil	Nil	Nil	Nil	1 × 10^2^	2 × 10^2^	≤2 × 10^2^
*E. coli*	Nil	Nil	Nil	Nil	Nil	Nil	Nil	Nil
*Salmonella*	Nil	Nil	Nil	Nil	Nil	Nil	Nil	Nil
*Bacillus*	Nil	Nil	Nil	Nil	Nil	Nil	Nil	Nil

^1^ For sample code (FTM50), refer to Table 1. FTM, foxtail millet; CFU, colony-forming units.

**Table 4:** Nutrient contribution of FTM50 noodles with respect to RDA ^1^.

Age Group (Years)	FTM50 Noodles													
	Energy (kcal)		Protein (%)		Phosphorous (mg/100 g)		Iron (mg/100 g)		Ca (mg/100g)		Na (mg/100g)		K (mg/100g)	
	464.62		14.78		536.75		5.02		23.41		28.29		38.15	
	Male													
	Reqd.	Fulfill	Reqd.	Fulfill	Reqd.	Fulfill	Reqd.	Fulfill	Reqd.	Fulfill	Reqd.	Fulfill	Reqd.	Fulfill
11–14	2500	18.58	45	32.84	1200	44.73	18	27.89	1200	1.95	2300	1.23	4500	0.85
15–18	3000	15.48	59	25.05	1200	44.73	18	27.89	1200	1.95	2300	1.23	4500	0.85
19–24	2900	16.02	58	25.48	1200	44.73	10	50.20	1200	1.95	2300	1.23	4700	0.81
25–50	2900	16.02	63	23.46	800	67.09	10	50.20	800	2.93	2300	1.23	4700	0.81
51+	2300	20.20	63	23.46	800	67.09	10	50.20	800	2.93	2300	1.23	4700	0.81
Age group (years)	Female													
11–14	2200	21.11	46	32.13	1200	44.73	15	33.47	1200	1.95	2300	1.23	4500	0.85
15–18	2200	21.11	44	33.59	1200	44.73	15	33.47	1200	1.95	2300	1.23	4500	0.85
19–24	2200	21.11	46	32.13	1200	44.73	15	33.47	1200	1.95	2300	1.23	4700	0.81
25–50	2200	21.11	50	29.56	800	67.09	15	33.47	800	2.93	2300	1.23	4700	0.81
51+	1900	24.45	50	29.56	800	67.09	10	50.20	800	2.93	2300	1.23	4700	0.81
Pregnant	2500	18.58	60	24.63	1200	44.73	30	16.73	1200	1.95	NA	NA	NA	NA
Lactating (1st six months)	2700	17.2	65	22.73	1200	44.73	15	33.47	1200	1.95	NA	NA	NA	NA
2nd six month	2700	17.2	62	23.83	1200	44.73	15	33.47	1200	1.95	NA	NA	NA	NA

^1^ For sample code, refer to Table 1. FTM, foxtail millet; RDA, recommended dietary allowance.

**Table 5:** Biochemical comparison of the FTM50 and commercial noodles ^1^.

Parameters	FTM50	A-1	A-2	A-3	A-4	A-5
Moisture (%)	6.61 ± 0.16 ^a^	3.67 ± 0.25 ^d^	4.87 ± 0.25 ^c^	5.40 ± 0.20 ^bc^	5.76 ± 0.15 ^b^	5.40 ± 0.20 ^bc^
Ash (%)	2.61 ± 0.16 ^a^	1.50 ± 0.10 ^b^	0.73 ± 0.11 ^d^	0.70 ± 0.10 ^d^	1.43 ± 0.15 ^bc^	1.13 ± 0.06 ^c^
Fat (%)	17.85 ± 0.08 ^c^	19.09 ± 0.08 ^b^	16.39 ± 0.05 ^d^	16.91 ± 0.27 ^d^	8.25 ± 0.50 ^e^	24.12 ± 0.12 ^a^
Fiber (%)	5.36 ± 0.04 ^a^	0.19 ± 0.02 ^b^	0.24 ± 0.04 ^b^	0.23 ± 0.05 ^b^	0.23 ± 0.04 ^b^	0.30 ± 0.04 ^b^
Protein (%)	14.78 ± 0.07 ^a^	4.42 ± 0.55 ^d^	5.18 ± 0.05 ^c^	9.50 ± 0.10 ^b^	9.10 ± 0.10 ^b^	9.13 ± 0.06 ^b^
Carbohydrate (%)	57.74 ± 0.09 ^c^	76.54 ± 4.64 ^ab^	77.98 ± 1.52 ^a^	66.74 ± 0.21 ^bc^	69.43 ± 5.63 ^ab^	69.83 ± 5.77 ^ab^
Energy (kcal/100 g)	464.61 ± 0.61 ^a^	446.12 ± 0.35 ^d^	441.98 ± 0.67 ^e^	458.92 ± 0.55 ^c^	422.56 ± 0.78 ^f^	460.94 ± 0.51 ^b^
Phosphorous (mg/100 g)	536.75 ± 0.08 ^a^	16.55 ± 0.42 ^d^	28.50 ± 0.66 ^c^	37.82 ± 0.36 ^b^	27.16 ± 1.20 ^c^	4.90 ± 0.65 ^e^
Iron (mg/100 g)	5.02 ± 0.54 ^c^	7.75 ± 0.18 ^b^	3.21 ± 0.22 ^d^	1.86 ± 0.03 ^e^	8.56 ± 0.11 ^a^	0.73 ± 0.06 ^f^
Calcium (mg/100 g)	23.40 ± 0.02 ^a^	0.93 ± 0.06 ^d^	1.29 ± 0.04 ^b^	0.93 ± 0.04 ^d^	0.74 ± 0.06 ^e^	1.07 ± 0.05 ^c^
Sodium (mg/100 g)	28.29 ± 0.43 ^e^	37.54 ± 0.33 ^d^	41.47 ± 0.12 ^c^	42.70 ± 0.32 ^b^	45.30 ± 0.17 ^a^	26.04 ± 0.07 ^f^
Potassium (mg/100 g)	38.15 ± 0.07 ^f^	155.62 ± 0.18 ^a^	132.55 ± 0.09 ^d^	153.77 ± 0.10 ^b^	134.72 ± 0.16 ^c^	90.72 ± 0.18 ^e^

^1^ Data present as mean ± standard deviation. This means that in a column with the same superscript, letters are not significantly different at (*p* < 0.05). For sample code, refer to Table 1. FTM, foxtail millet; A-1 to A-5, commercial noodles available in Bangladesh market.

## Data Availability

Data is available within the article.

## References

[1] Durand-Morat A., Bairagi S. (2022). International Rice Outlook: International Rice Baseline Projections 2021—2031.

[2] Abedin M.J., Abdullah A.T.M., Satter M.A., Farzana T. (2022). Physical, Functional, Nutritional and Antioxidant Properties of Foxtail Millet in Bangladesh. Heliyon.

[3] Meherunnahar M., Chowdhury R.S., Hoque M.M., Satter M.A., Islam M.F. (2018). Comparison of Nutritional and Functional Properties of BK2 Foxtail Millet with Rice, Wheat and Maize Flour. Progress. Agric..

[4] Alzand K.I., Bofaris M.S.M., Ugis A. (2019). Chemical Composition and Nutritional Value of Edible Wild Growing Mushrooms: A Review. World J. Pharm. Res..

[5] Tufail T., Ain H.B.U., Saeed F., Nasir M., Basharat S., Rusu A.V., Hussain M., Rocha J.M., Trif M., Aadil R.M. (2022). A Retrospective on the Innovative Sustainable Valorization of Cereal Bran in the Context of Circular Bioeconomy Innovations. Sustainability.

[6] Washingali D.C. (1990). Official Methods of Analysis.

[7] Yu A.H.M., Phoon P.Y., Ng G.C.F., Henry C.J. (2020). Physicochemical Characteristics of Green Banana Flour and Its Use in the Development of Konjac-Green Banana Noodles. J. Food Sci..

[8] American Association of Cereal Chemists (2000). Approved Methods of the American Association of Cereal Chemists.

[9] Salfinger Y., Tortorello M.L., Tortorello M.L., Salfinger Y. (2015). Compendium of Methods for the Microbiological Examination of Foods.

[10] Sutton S. (2010). The Most Probable Number Method and Its Uses in Enumeration, Qualification, and Validation. J. Valid. Technol..

[11] ChauhaN D., Kumar K., Kumar S., Kumar H. (2018). Effect of Incorporation of Oat Flour on Nutritional and Organoleptic Characteristics of Bread and Noodles. Curr. Res. Nutr. Food Sci. J..

[12] Jamroz D., Wiliczkiewicz A., Wertelecki T., Orda J., Skorupińska J. (2005). Use of Active Substances of Plant Origin in Chicken Diets Based on Maize and Locally Grown Cereals. Br. Poult. Sci..

[13] FAO (2022). Food and Agriculture Organization of the United Nations.

[14] Hidvégi M., Békés F. (1985). Mathematical Modeling of Protein Nutritional Quality from Amino Acid Composition. Amino Acid Composition and Biological Value of Cereal Proteins.

[15] Zinina O., Merenkova S., Rebezov M., Tazeddinova D., Yessimbekov Z., Vietoris V. (2019). Optimization of Cattle By-Products Amino Acid Composition Formula. Agron. Res..

[16] Kaur R., Prasad K. (2022). Effect of Malting and Roasting of Chickpea on Functional and Nutritional Qualities of Its Protein Fractions. Int. J. Food Sci. Technol..

[17] Niu M., Hou G.G. (2019). Whole Wheat Noodle: Processing, Quality Improvement, and Nutritional and Health Benefits. Cereal Chem..

[18] Hutabarat D.J.C., Bowie V.A. Bioactive Compounds in Foxtail Millet (*Setaria Italica*)-Extraction, Biochemical Activity, and Health Functional: A Review. Proceedings of the IOP Conference Series: Earth and Environmental Science.

[19] Pandey P., Malagi U., Yenagi N., Fatima A. (2017). Optimization of Value Added Vermicelli Based on Foxtail Millet (*Setaria Italica*). Int. J. Pure App. Biosci..

[20] Chillo S., Laverse J., Falcone P.M., Protopapa A., DelNobile M.A. (2008). Influence of the Addition of Buckwheat Flour and Durum Wheat Bran on Spaghetti Quality. J. Cereal Sci..

[21] Sá A.G.A., Moreno Y.M.F., Carciofi B.A.M. (2020). Food Processing for the Improvement of Plant Proteins Digestibility. Crit. Rev. Food Sci. Nutr..

[22] Mahmoud E.A.M., Nassef S.L., Basuny A.M.M. (2012). Production of High Protein Quality Noodles Using Wheat Flour Fortified with Different Protein Products from Lupine. Ann. Agric. Sci..

[23] Oser B.L. (1959). An Integrated Essential Amino Acid Index for Predicting the Biological Value of Proteins. Protein Amin. Acid Nutr..

[24] Satter M., Ara H., Jabin S., Abedin N., Azad A.K., Hossain A., Ara U. (2014). Nutritional Composition and Stabilization of Local Variety Rice Bran BRRI-28. Int. J. Sci. Technol..

[25] Srinivasan D., Parkin K., Fennema O. (2008). Fennema’s Food Chemistry.

[26] DiCostanzo M., DePaulis N., Capra M.E., Biasucci G. (2022). Nutrition during Pregnancy and Lactation: Epigenetic Effects on Infants’ Immune System in Food Allergy. Nutrients.

[27] Mounika D., Sangeetha U., Sireesha G. (2022). Estimation of Phytochemicals in Millets and Selected Millet Products. Indian J. Appl. Pure Bio..

[28] Witkoś J., Hartman-Petrycka M. (2022). The Use of Dietary and Protein Supplements by Women Attending Fitness Clubs on a Recreational Basis and an Analysis of the Factors Influencing Their Consumption. Cent. Eur. J. Sport Sci. Med..

[29] Arya C., Bisht A. (2022). Small Millets: Path to Food and Nutrition Security. Small Millet Grains: The Superfoods in Human Diet.

[30] Meherunnahar M., Hoque M., Islam M. (2020). New Bari Wheat Cultivars: Evaluation of Processing and Nutrition Value. IJISRT Com..

[31] Islam M., Akash S., Jony M.H., Nowrin F.T., Rahman M., Rauf A., Thiruvengadam M. (2023). Exploring the Potential Function of Trace Elements in Human Health: A Therapeutic Perspective. Mol. Cell. Biochem..

